# Hypercholesterolemia and COVID-19: Statins for Lowering the Risk of Venous Thromboembolism

**DOI:** 10.3389/fcvm.2021.711923

**Published:** 2021-10-13

**Authors:** Alpo Vuorio, Riitta Lassila, Petri T. Kovanen

**Affiliations:** ^1^Department of Forensic Medicine, University of Helsinki, Helsinki, Finland; ^2^Mehiläinen Airport Health Centre, Vantaa, Finland; ^3^Research Program Unit in Systems Oncology, Coagulation Disorders Unit, Helsinki University Hospital, University of Helsinki, Helsinki, Finland; ^4^Wihuri Research Institute, Biomedicum Helsinki 1, Helsinki, Finland

**Keywords:** COVID-19, statins, venous thromboembolism, familial hypercholesterolemia, LDL cholesterol

## Introduction

In this Opinion letter, we focus on the prevention of venous thromboembolism in hypercholesterolemic patients suffering from COVID-19. We present the heterozygous form of familial hypercholesterolemia (HeFH) as the prototype of genetically determined pure hypercholesterolemia. Moreover, we examine the special therapeutic challenges that one faces when treating a COVID-19 patient suffering from the common (non-HeFH) form of hypercholesterolemia.

HeFH is an autosomal dominant disorder resulting in a lifelong two- to three-fold elevation of serum low-density lipoprotein cholesterol (LDL-C) (1, 2). According to a recent report including 62 studies and over 7 million individuals, the overall prevalence of HeFH is about 1:300 among the general population worldwide (3, 4). Thus, roughly 30 million people suffer from HeFH. Most importantly, severe hypercholesterolemia leads to about 10-fold increased risk for premature atherosclerotic cardiovascular disease (ASCVD), premature atherothrombotic complications being the leading cause of mortality in this disease (5). In addition to the highly elevated serum LDL-C, patients with HeFH tend to have serum lipoprotein(a) [Lp(a)] levels that are higher than in the general population (6).

The Lp(a) particles are LDL-like lipoprotein particles in that they carry cholesterol which contributes to cholesterol accumulation in the developing atherosclerotic lesions. Moreover, the Lp(a) particles carry proinflammatory oxidized phospholipids and thus contributing also to the inflammatory reactions of the atherosclerotic arterial wall, including promotion of endothelial dysfunction (7). Since the plasma level of Lp(a) particles is genetically determined, an increased level constitutes a life-long atheroinflammatory burden to the arterial wall. Finally, the Lp(a) particles are prothrombotic since they activate endothelial cells and platelets and impair spontaneous fibrinolysis of a developing thrombus (6, 8, 9).

Continuous and effective blood cholesterol-lowering therapy increases the life expectancy of the HeFH patients approximately to the level of the general population (10). Effective LDL-C-lowering with a statin should be started in children 8–10 years old (11). Moreover, multidrug pharmacotherapy in which ezetimibe or a PCSK9 inhibitor is added to the statin monotherapy is deemed safe and effective. When needed to reach an effective LDL-C lowering, such combination therapy could be initiated already in adolescent HeFH patients (11–14).

## COVID-19 and Venous Thromboembolism

In a recent meta-analysis involving hospitalized patients with COVID-19, the incidence of deep vein thrombosis (DVT) was 11.8% (7.1–17.4) (95%, CI), and that of pulmonary embolism (PE) was 13.5% (8.4–29.5) (95%, CI) (15). In a later French nationwide retrospective cohort study, which included 89 530 hospitalized COVID-19 patients, the incidence of VTE (without or with PE) was 4.9% and that of PE 3.4%, these incidences being much higher than those for patients hospitalized, e.g., for seasonal influenza (16).

Patients with CHD carry, in general, a higher risk for VTE than patients without CHD (17). However, among critically ill COVID-19 patients, only limited data are available on the coexisting risk of VTE and CHD. Thus, in a study of critically ill patients with COVID-19 and having thromboprophylaxis, only 2 of 88 patients had CHD, and both belonged to the group which had developed VTE (*N* = 40) (18). Of note, overweight, hypertension and diabetes are additional risk factors for VTE in patients with COVID-19. Furthermore, the incidence of COVID-19-associated arterial thrombosis involving the coronary, cerebrovascular, and peripheral arteries was reported to be around 4% (19). Although we have learned to improve and intensify the thromboprophylactic strategies after the first waves of the pandemic, the above studies show that despite such efforts, a risk for thrombotic and thromboembolic complications still prevails.

## Statins and COVID-19

Ever-increasing numbers of evidence-based data derived from individual studies and meta-analyses show that statins are beneficial for the prognosis of COVID-19 patients and, accordingly, a consensus expert statement calls for a continuation of statin treatment in COVID-19 patients also after the illness (20–24). The amenable effect of statins especially applies to patients with HeFH because of both the magnitude and duration of the elevation of serum LDL-C, which often combines with an elevated Lp(a) level and then jointly correlates with the severity of systemic endothelial dysfunction and coagulation propensity (25). Of note, however, statins fail to lower Lp(a) levels.

Statins reduce thrombin and fibrin formation (26–28). Indeed, in many epidemiological studies, this class of drugs has been shown to prevent thrombosis. The preventive action of statins also applies to the COVID-19-affected venous system and the development of PE, which themselves pose additional significant threats in severe COVID-19 on top of the microvascular damaging effect of the SARS-CoV-2 infection (29). In addition, the anti-inflammatory effects of statins have been shown in a cytokine interaction model involving cocultured human muscle and mononuclear cells (30). Especially among the high-risk HeFH patients, statin-induced improvement of endothelial function may decrease the incidence of both arterial thrombosis, typically manifested as acute myocardial infarction (AMI), and VTE (31, 32).

## Familial Hypercholesterolemia and Venous Thromboembolism

Since HeFH (like in other hypercholesterolemias), LDL-C accumulates in the thick subendothelial inner layers of the arterial walls but not in the thin subendothelial layers of veins, it is evitable that the atherosclerotic changes in the arterial system determine the clinical outcome in this disease. However, since high levels of LDL-C and Lp(a) cause endothelial dysfunction throughout the vascular system, including the venous endothelium, thrombotic and thromboembolic clinical events may also ensue. Yet, data on whether the HeFH patients, in general, have an increased risk for VTE are minimal, one likely reason being the fact that, in general, HeFH remains heavily underdiagnosed throughout the world (33). This apparently puzzling issue remains a challenge for future studies because in the general population hypercholesterolemia is a risk factor for VTE (34), and statins show primary prevention effects on VTE (35). In the study by Kawasaki et al. (34), including 109 patients with DVT and 109 controls, even modest elevations of serum total cholesterol level (equal to or above 220 mg/dl or 5.7 mmol/l) did associate with an elevated risk of DVT (OR 2.6). Therefore, we may surmise that in untreated HeFH patients, the exceptionally high cholesterol levels will damage the venous endothelium.

Krogh et al. (36) reported that in HeFH, the prevalence of VTE was ~14% among 60-year-old male and female patients, and according to calculations the annual incidence rate is 2/1,000. Based on the systematic review of VTE, the incidence of VTE among the 60-year-old HeFH patients is close to that in 10–20 years older persons in the general population [26]. Accordingly, the severe hypercholesterolemia observed in untreated or undertreated HeFH patients, i.e., in the majority of HeFH patients, increases the risk of VTE. Given that globally the majority of HeFH patients remains undiagnosed, and even if diagnosed, they remain undertreated (33), we can infer that the vast majority—if not all—of the HeFH patients with COVID-19 carry an elevated risk of VTE. Provided the hypercholesterolemia of an HeFH patient is accompanied by other systemic endothelial-damaging risk factors, such as hypertension, hyperglycemia, or obesity, the risk of VTE must be still increased.

We have recently pointed to the potentially beneficial effects of cholesterol-lowering therapy in preventing endothelial dysfunction and thrombotic events in HeFH patients with COVID-19, particularly when a statin is combined with a PCSK9 inhibitor (37). Regarding the thrombotic and thromboembolic risk, it is important to note that the cholesterol-lowering drugs also decrease the turnover of fibrin, i.e., the statins decrease D-dimer levels by about 15%, and the PCSK9 inhibitors decrease the plasma level of the anti-fibrinolytic Lp(a) particles by about 30% (38, 39). The Lp(a)-lowering effect of a PCSK9 inhibitor appears to prevent recurring acute coronary syndrome (ACS) in patients on maximally tolerated statin therapy which has already resulted in a low LDL-C level (around 70 mg/dl or 1.8 mmol/l) (40). Therefore, we advocate using a PCSK9 inhibitor in hospitalized HeFH patients with COVID-19, particularly those with an elevated Lp(a) level and a history of an ACS.

## Antiplatelet Drugs

A recent meta-analysis was carried out to find the effect of antiplatelet treatments on patients with COVID-19 infection (41). In this analysis, the treatments included acetylsalicylic acid and P2Y12 inhibitors. The meta-analysis included nine articles and 5,970 patients, and it showed that the use of the studied antiplatelet drugs did not associate with a reduced risk for the severe form of COVID-19 (OR = 0.98, 95% CI: 0.64–1.50, *p* = 0.94; *I*^2^ = 65%). This result resembles that obtained in a Japanese cohort study of a total of 4,265 hospitalized patients (42). In this latter analysis, the use of antiplatelet drugs failed to impact the severity of COVID-19.

Anticoagulants have been more potent in inhibiting both venous thrombosis and fibrin formation in critical vessels, while antiplatelet agents alone seem unable to control the viral procoagulant action. Statins, with their multiple calming-down influences, which occur *via* their anti-inflammatory and especially *via* their tissue factor synthesis-reducing actions, provide a multitargeted adjunct therapeutic opportunity (28). However, any clinical meta-analysis faces the problem of pooling various risk category patients together, which, in the case of trying to balance the bleeding and thrombosis risks, may neutralize the personalized needs and benefits of a medication. The highest risk patient would likely benefit most from a combination therapy involving both an antiplatelet drug and a statin, in addition to thromboprophylactic doses of low-molecular-weight heparin (43). Some cardiovascular patients, and even those with subclinical atherosclerosis, also have atrial fibrillation, which often requires anticoagulation (44). Accordingly, statins need to be used in association with anticoagulation, at least in these high-risk hypercholesterolemic patients. In coagulation, the mechanisms of thrombin formation are synergistic rather than additive. Therefore, the inhibition of coagulation calls upon combination strategies to alleviate thrombotic complications in the hospitalized high-risk HeFH patients with COVID-19. The further role of low-molecular-weight heparin, at the latest during hospital admission, would allow protection against cardiovascular complications (45, 46).

## Concluding Remarks

Given the increased baseline incidence of VTE in HeFH due to the presence of a lifelong severe hypercholesterolemia, the benefits gained by efficient cholesterol-lowering therapy in the HeFH patients are likely to exceed that of the patients with a diet-induced hypercholesterolemia, in whom statin use is also advocated. Indeed, observational population-based cohort studies show that statin treatment decreases recurrent VTE, which carries an especially high reoccurrence rate of around 15–20% (47, 48). Another interesting piece of information on the favorable effect of cholesterol-lowering therapy comes from the recent *post-hoc* analysis of the FOURIER and ODYSSEUS OUTCOMES (49). In this analysis, a 31% relative risk reduction of VTE with PCSK9 inhibition [HR, 0.69 (95% CI, 0.53–0.90); *p* = 0.017] was observed. The risk reduction particularly applied to carriers of high serum Lp(a) who benefitted from the PCSK9 treatment to prevent recurrent VTEs.

Overall, an increased risk of bleeding complications occurs in about 5–8% of the COVID-19 patients (50). Thus, statin and PCSK9 inhibitor treatment for the lowering of LDL-C levels, when used together with a strong antithrombotic medication, may impair hemostasis and cause intracerebral bleeds which are mostly fatal in COVID-19 (51, 52). Based on the above considerations, the use of these medications should be personalized to safely master the critical and delicate balance between the unphysiological coagulation of thrombosis and bleeding tendency in an individual COVID-19 patient. This analysis is based on the medical history of the patient and his family, potentially untreated hypertension, actual lipid levels, hepatic (prolonged prothrombin time) and renal impairment, and an analysis of relevant coagulation laboratory tests and exclusion of anemia and thrombocytopenia, which together with low fibrinogen and von Willebrand factor (usually high in COVID-19) levels refer to a bleeding risk.

Several reasons support the use of efficient cholesterol-lowering treatment in HeFH patients with COVID-19. The pre-hospitalization use of statins has recently been observed to reduce mortality in COVID-19 by more than 50% (20). We wish to highlight the extra benefit of statin use in the prevention of potential venous thrombotic events. So far, continued cholesterol lowering in hospitalized HeFH with COVID-19 has been suggested. It seems to be justified considering the enhancement of cholesterol-lowering medication in these patients also after discharge from the hospital, as COVID-19 may cause a long-term increased cardiovascular risk on top of the preexisting high cardiovascular risk in HeFH patients (19, 53, 54). While the specific information on the role of cholesterol-lowering therapy in the VTE risk is being gathered, effective means to lower plasma cholesterol levels are likely to be paramount for improved prognosis among HeFH patients with COVID-19.

Finally, combining cholesterol-lowering and antithrombotic medications when attempting to prevent the often-lethal thrombotic complications of COVID-19 is prudent. This particularly applies to statins and, on a discretional basis, also to the PCSK9 inhibitors, which possess a combined LDL-C- and Lp(a)-lowering effect, thereby gaining potentially unique antithrombotic effects that inhibit both coagulation and platelet activation (28, 55). In this way, both statins and PCSK9 inhibitors are valuable adjuncts to the overall antithrombotic armamentarium in hospitalized hypercholesterolemic COVID-19 patients, particularly in COVID-19 patients with severe hypercholesterolemia—whether or not they have the diagnosis of HeFH. In these patients, for short-term and long-term prophylaxis against thrombosis, the treating physicians need to bear in mind not only the traditional preventive target vessels, i.e., those in the arterial system, but also those in the venous system, and without forgetting the risks of bleeding complications. Thus, any individual treatment decision needs to be made under the vigilance of the delicate hemostatic balance.

## Author Contributions

AV and PK: writing the first draft. AV, RL, and PK: editing to produce the final draft. All authors contributed to the article and approved the submitted version.

## Conflict of Interest

PK has received lecture honoraria and/or travel fees from Amgen, Novartis, Raisio Group, and Sanofi. RL has received lecture honoraria and served in advisory boards of Alexion, Astra Zeneca, Bayer, Pfizer, Roche, and Sanofi. The remaining author declares that the research was conducted in the absence of any commercial or financial relationships that could be construed as a potential conflict of interest.

## Publisher's Note

All claims expressed in this article are solely those of the authors and do not necessarily represent those of their affiliated organizations, or those of the publisher, the editors and the reviewers. Any product that may be evaluated in this article, or claim that may be made by its manufacturer, is not guaranteed or endorsed by the publisher.
